# Effect of Long-Term Voluntary Exercise Wheel Running on Susceptibility to Bacterial Pulmonary Infections in a Mouse Model

**DOI:** 10.1371/journal.pone.0082869

**Published:** 2013-12-23

**Authors:** Pauline B. van de Weert – van Leeuwen, Angélica M. M. de Vrankrijker, Joachim Fentz, Oana Ciofu, Jørgen F. P. Wojtaszewski, Hubertus G. M. Arets, Hendrikus J. Hulzebos, Cornelis K. van der Ent, Jeffrey M. Beekman, Helle K. Johansen

**Affiliations:** 1 Department of Pediatric Pulmonology, University Medical Centre Utrecht, Utrecht, The Netherlands; 2 Department of Translational Immunology, University Medical Centre Utrecht, Utrecht, The Netherlands; 3 Centre for Molecular and Cellular Intervention, University Medical Centre Utrecht, Utrecht, The Netherlands; 4 Department of International Health, Immunology and Microbiology, Panum Institute, University of Copenhagen, Copenhagen, Denmark; 5 Department of Clinical Microbiology, Rigshospitalet, Copenhagen, Denmark; 6 Department of Nutrition, Exercise and Sports, Section of Molecular Physiology, University of Copenhagen, Copenhagen, Denmark; 7 Child Development & Exercise Centre, University Medical Centre Utrecht, Utrecht, The Netherlands; University of Buenos Aires, Cardiovascular Pathophysiology Institute, Argentina

## Abstract

Regular moderate exercise has been suggested to exert anti-inflammatory effects and improve immune effector functions, resulting in reduced disease incidence and viral infection susceptibility. Whether regular exercise also affects bacterial infection susceptibility is unknown. The aim of this study was to investigate whether regular voluntary exercise wheel running prior to a pulmonary infection with bacteria (*P. aeruginosa*) affects lung bacteriology, sickness severity and phagocyte immune function in mice. Balb/c mice were randomly placed in a cage with or without a running wheel. After 28 days, mice were intranasally infected with P. *aeruginosa*. Our study showed that regular exercise resulted in a higher sickness severity score and bacterial (*P. aeruginosa*) loads in the lungs. The phagocytic capacity of monocytes and neutrophils from spleen and lungs was not affected. Although regular moderate exercise has many health benefits, healthy mice showed increased bacterial (*P. aeruginosa*) load and symptoms, after regular voluntary exercise, with perseverance of the phagocytic capacity of monocytes and neutrophils. Whether patients, suffering from bacterial infectious diseases, should be encouraged to engage in exercise and physical activities with caution requires further research.

## Introduction

It has been shown that regular exercise is positively associated with health. It improves muscle strength and function, cardiorespiratory fitness, quality of life and has been suggested to affect immune function as well. However, immune modulatory effects induced by regular exercise remain poorly studied [1]–[4]. Regular exercise of moderate intensity has been shown to exert anti-inflammatory effects (e.g. in obesity, atherosclerosis, diabetes) and may also improve immune effector functions, resulting in reduced disease incidence and viral infection susceptibility. The opposite has been observed for prolonged or very intense exercise [1]; [2]; [4]–[8]. In animal models it was shown that a period of moderate regular exercise reduced microbial load, inflammation, morbidity and mortality upon a viral infection [9]–[12]. Recently, a large longitudinal cohort study in 1002 healthy adults showed that the number of days with upper respiratory tract infections (URTIs) was significantly reduced in physically fit and active adults, with higher numbers in people that hardly, or intensively exercised [13]. These studies suggest that specific exercise programs may be used to modify the course of inflammatory and/or infectious diseases.

The effects of regular exercise to microbial loads, morbidity and mortality upon a bacterial infection are unknown. In this study, we focused on *Pseudomonas aeruginosa* (*P. aeruginosa*), which is a gram-negative pathogen. *P. aeruginosa* is the most frequently isolated pathogen in patients with nosocomial acquired pneumonia, nosocomial acquired burn-wound infections [14] and pulmonary infections in patients with cystic fibrosis (CF) [15]–[17]. The aim of this study was to investigate whether regular voluntary exercise wheel running prior to a pulmonary infection with *P. aeruginosa* affects lung bacteriology, sickness severity and phagocyte immune function in mice.

## Materials and Methods

### Animals and ethics

Female Balb/c mice (n = 40, 12–15 weeks old) were obtained from Taconic (Tornbjerg, Denmark). The study procedure was approved by the local animal care committee (Panum Institute, Copenhagen, Denmark) and The Animal Experiments Inspectorate (2008/561–754). Mice were housed in a pathogen-free experimental unit with barrier provisions and received commercial food and water ad libitum.

### Study procedure

**Figure 1:**
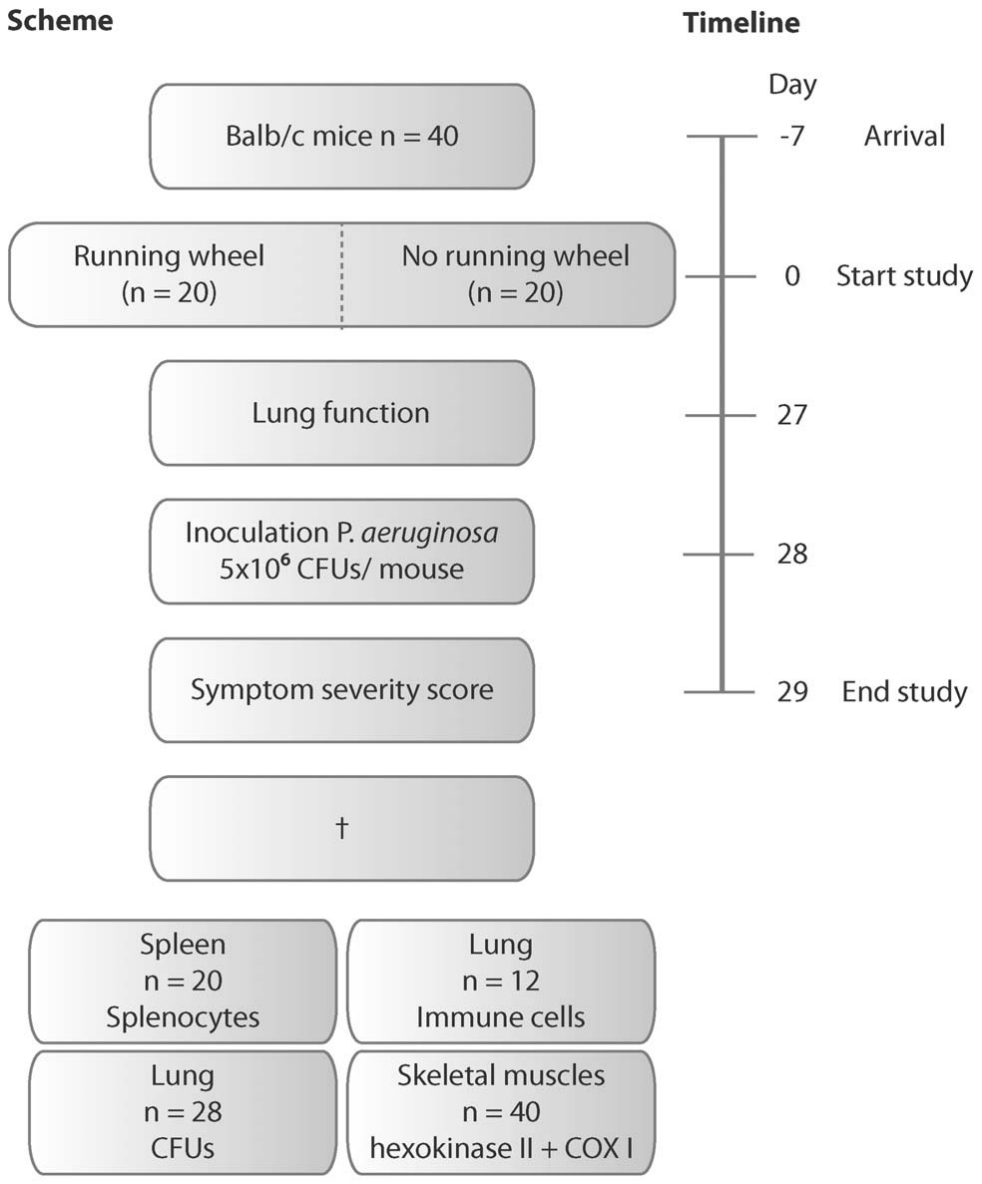
Schematic representation of the study procedure.

Following 1 week recovery upon arrival, mice were randomly placed in individual cages, which were supplied with (N = 20) or without a running wheel (N = 20) (Techniplast activity cage, wheel Ø: 23 cm; Techniplast, Buguggiate, Italy). Mice in the running wheel group had free access to the activity wheel for 28 days. Distance covered weekly in the running wheel was measured online by a cycle computer (BC 1400; Sigma Sport, Neustadt, Germany). General health monitoring was performed daily and lung function was measured at day 27. At day 29 mice were inoculated intranasally with *P. aeruginosa* (**Fig. 1**), after anaesthetization using an intraperitoneal injection of a mixture of 65 mg/kg ketamine (Intervet, Skovlunde, Denmark), 13 mg/kg Xylazine (Intervet) in sterile isotonic saline. The bacterial inoculum (50 μL sterile saline containing 5×10^6^ colony forming units (CFU) of *P. aeruginosa*) was applied drop wise to the nostrils of the mice. This dose was chosen, based on previous titration studies, which showed a small possible working dose of 1×10^6^ to 1×10^7^ CFUs/50 µL. Lower doses were fully cleared by all mice and higher doses were lethal within a few hours. Mice were held in an upright position until the complete inoculum was inhaled. Mice had access to the running wheels until the inoculation took place, which represents the usual sequence of events best. In normal life, patients do not know in advance when *P. aeruginosa* acquisition will take place. Exercise will therefore be continued until patients get sick. Following inoculation, mice were housed in an isolated cabinet (Scanbur, Karlslunde, Denmark) and had no access to a running wheel anymore. 16 hours post-infection (overnight), following symptom severity scoring, mice were sacrificed using 300 µl of an intraperitoneal injection containing 200 mg/ml pentobarbital and 20 mg/ml lidocaine, since mice were too sick to let them live. A mortality study was not allowed by the ethical committee.

### Measurement of Pulmonary Function

Whole Body Plethysmography (Buxco, Troy, NY, USA) was performed as previously described (n = 40) [18]. In brief, WBP was used to measure the effect of the intervention on lung function in mice at day 27. Mice were individually placed inside the chambers. During a 5 minute measurement, breathing frequency (breaths/min) and tidal volume (ml) were measured and recorded.

### Bacteria and inoculation

Mice were inoculated using the laboratory *P. aeruginosa* strain PAO1 [19]. Bacteria were grown overnight in Luria–Bertani (LB) medium at 37°C 175 rpm. The next day, 1 ml of broth was resuspended in 20 ml of fresh LB broth and allowed to grow until the mid-logarithmic phase (OD of 0.6 at 600 nm ≈1×10^9^ cells/ml). Bacteria were washed and resuspended in 0.9% sterile saline at 1×10^8^ CFUs/ml (5×10^6^ CFUs/50 μL). Number of CFUs in inocula was verified by plating serial dilutions on blue agar plates (a modified Conradi Drigalski's medium selective for Gram-negative rods; State Serum Institute, Copenhagen, Denmark) overnight at 37°C. The inoculum dose of 5×10^6^ CFUs/50 µl was based on previous pilot experiments, which showed full clearance of the bacteria when ≤1×10^6^ CFUs/50 µl were administered, whereas a dose ≥1×10^7^/50 µl was lethal (data not shown). For the phagocytosis assay, an EGFP-labeled PAO1 strain was used, which has been described previously [20]. Bacteria were grown overnight in Luria–Bertani (LB) medium containing 100 µg/ml ampicillin and 10 µg/ml kanamycin, at 37°C 175 rpm. The next day, 1 ml broth was resuspended in 20 ml of fresh LB broth and allowed to grow until the mid-logarithmic phase (OD of 0.6 at 600 nm ≈1×10^9^ cells/ml). Bacteria were washed and diluted in PBS until a final concentration of 2×10^7^ CFUs/ml was reached.

### Symptom severity score

Mice (n = 40) were scored for symptom severity twice 16 hours following inoculation with *P. aeruginosa* by an investigator, who was blinded for the experimental conditions. Symptom severity score contained typical symptoms of illness, which was adapted from Murphy et al. (Table 1) [11]. Mice that displayed any of these symptoms were considered as morbid. Cumulative scores ranged from 0 to 10, based on the varying degree of symptoms of sickness.

**Table 1:** Symptom severity scoring system.

Eyes	0– no signs, normal
	1– sore red eyes
Lesions	0– none
	1– lesion on head
Fur	0– well groomed
	1– ruffled fur
Neurological/neuromuscular	0– normal movement
	2– hunched back
	2– hind limb paralysis
	3– unresponsiveness

Symptom severity scoring system was adapted from Murphy et al.^11^. Animals were scored twice post-infection. Cumulative score may range from 0 to 10, indicating no to severe illness.

### Isolation of immune cells from spleen and lung

Immune cells were harvested from sacrificed mice, 16 hours following inoculation with *P. aeruginosa*. Spleens (n = 20 of n = 40 mice) and lungs (n = 24 of n = 40 mice) were incubated in 0.9% NaCl and placed on ice. Explants were homogenized using a cell-strainer (100 μM, BD Biosciences, NJ, USA) in RPMI 1640 wash medium supplied with 2% FCS, 1% L-glutamin and Penicillin (100 U/ml) and Streptomycin (100 μg/ml). Before further handling, erythrocytes were removed by an erythrocyte lysis buffer Hybri-Max R7757, according to the manufacturer's protocol. Next, immune cells (splenocytes and immune cells from the lung) were washed in RPMI 1640 wash medium and frozen in freeze medium, which contained 90% FCS and 10% DMSO. Media and supplements were obtained from Invitrogen (Invitrogen, CA, USA).

### Lung bacteriology

Lungs were removed from sacrificed mice, 16 hours following inoculation with *P. aeruginosa*. Lungs (n = 28 of n = 40 mice) were incubated in 0.9% NaCl and placed on ice. Both lungs of individual mice were pooled and homogenized on ice in 3 ml sterile saline using a homogenizer (Heidolph diax 600, Struers, Ballerup, Denmark) at 13500 rpm. To determine the amount of CFUs, serial dilutions were plated on blue agar plates and incubated overnight at 37°C. Colonies were tested for oxidase activity with oxydase reagens (State Serum Institute). Colonies attained a deep blue colour within 10 sec when they were of *P. aeruginosa* origin (presence of the enzyme complex cytochrome c).

### Determination of skeletal muscle mitochondrial enzymes

#### Muscle lysate preparations

The white part of the musculus (m.) gastrocnemius and m. quadriceps femoris were removed from sacrificed mice and immediately frozen in liquid nitrogen and stored at −80°C. Whole cell lysates were prepared by homogenization in 2 ml Eppendorf tubes using a Polytron (PT 1200, Kinematica) and were incubated in ice-cold lysis buffer A [50 mM HEPES (pH 7.4) 10% glycerol, 20 mM Na Pyrophosphate, 150 mM NaCl, 1% NP-40, 20 mM β-glycerophosphate, 10 mM NaF, 1mM EDTA, 1 mM EGTA, 2 mM PMSF, 10 µg/ml aprotinin, 10 µg/ml leupeptin, 2 mM Na3VO4, 3 mM benzamidine] for 20 sec (PT 3100; Kinematica). Homogenates were rotated end-over-end at 4°C for 1 h. Soluble debris was collected by centrifugation at 17.500 g (30 min, 4°C) and supernatants were snapfrozen in liquid nitrogen and stored at −80°C.

#### SDS-PAGE and Western Blotting

Total protein content in lysates were determined by the bicinchoninic acid method (Pierce Chemical, Rockford, IL). Muscle whole cell lysates were subjected to SDS-page (7.5–15% Tris•HCl gels; Criterion, Bio-Rad, Denmark), and were transferred (semidry) to PVDF membranes (Immobilion Transfer Membrane, Milipore), blocked with Tris-buffered saline-Tween 20 (10 mM tris-Base (pH 7.4), 0.9% NaCl, 1% Tween 20 (TBST) +2% skimmed milk), and incubated with primary antibodies (TBST +2% skimmed milk) followed by incubation with horseradish peroxidase-conjugated secondary antibody (TBST +2% skimmed milk) (Dako, Glostrup, Denmark). Primary antibodies used were: anti-hexokinase II (anti-HK II) and anti cytochrome C Oxidase I (Molecular Probes, Eugene, OR). Bands were visualized after washing of membranes and incubation with chemiluminescent horseradish peroxidase substrate (Millipore, Immobilon, Denmark) using a CCD-camera (Kodak Image Station 2000M, Denmark).

### Phagocytosis

Isolated immune cells from lung or spleen were thawed using RPMI 1640 wash medium supplemented with 10% FCS. We have already shown in human phagocytes that the freeze-thawing process does not affect the phagocytosis capacity of immune cells (data not shown). Cells were washed with RPMI 1640 wash medium supplemented with 2% FCS. The EGFP-labelled PAO1 strain was opsonized for 30 min by 4% mouse serum (Dako, Heverlee, Belgium). Next, immune cells (1×10^6^/50 µl) were incubated with opsonized PAO1 (1×10^7^/50 µl) for 30 minutes. The infection ratio of 1∶10 and amount of serum used was based on previous work of others [21]; [22] and our own work in human phagocytes and PAO1 (unpublished data). A small pilot study in mouse phagocytes gave similar results to what we observed in the phagocytosis assay with human phagocytes (data not shown). To stop phagocytosis, cells were placed on ice and 100 μL cold paraformaldehyde 4% (PFA; Klinipath, Duiven, Netherlands) was added immediately and incubated for 30 min at 4°C. The phagocytic capacity (percentage of EGFP-positive cells) was measured using flow cytometry. Cell populations were identified using forward and side scatter. Identification of populations using CD-marker specific stainings, prior to the start of the phagocytosis assay, interferes with the phagocytosis capacity. CD14, for example, can be used for identification of monocyte populations, but is also needed for recognition of LPS, which stimulates phagocytosis upon binding. Recognition of CD-marker epitopes post phagocytosis is hampered, because immune cells are fixated using formaldehyde. Furthermore, extra washing and spinning procedures would further continue phagocytosis processes, although cells are fixated and kept on ice.

### Statistical analysis

A Kolmogorov-Smirnov test was used to test whether the variables were normally distributed. Data were expressed as means ± standard errors of the mean (SEM) and analyzed using an unpaired Student's T-test when variables were normally distributed, otherwise as median ± interquartile range (IR) and a Mann Whitney U-test. A p-value <0.05 was considered as statistically significant. All data were analyzed in SPSS statistics version 20.0 for Windows.

## Results

### Effectiveness of voluntary exercise wheel running

**Figure 2:**
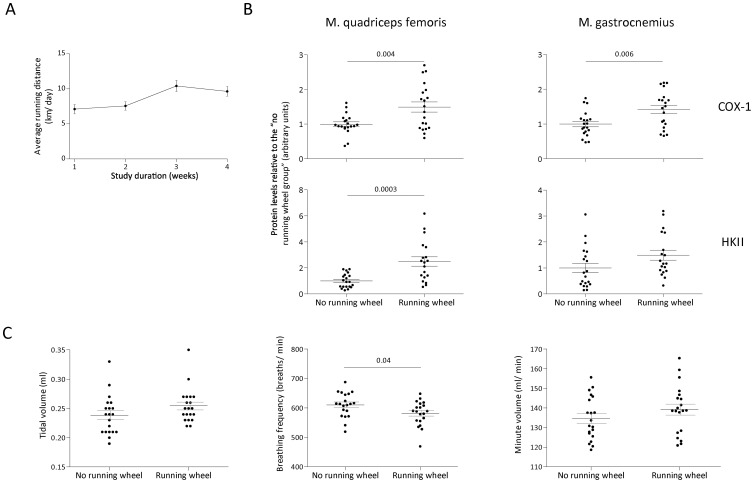
Effectiveness of voluntary exercise wheel running. Each dot represents one mouse. **A**. Mean ± SEM running distance per day per study week (n = 20). **B**. Effect of exercise wheel running on HK II and COX I protein levels in the white part of the m. quadriceps and m. gastrocnemius (n = 20 per group). Protein contents were expressed relative to the “no running wheel group” (Mean ± SEM). **C**. Effect of exercise wheel running on lung function (Mean ± SEM): tidal volume (ml), breathing frequency (breaths/min) and minute volume (ml/min) (n = 20 per group).

To assess whether voluntary exercise wheel running was adequate to achieve training-induced adaptations, daily running distance and HK II and COX I skeletal muscle protein levels were determined in the white part of the m. quadriceps femoris and m. gastrocnemius. On average mice ran 8.7±0.1 km per day (mean ± SEM) (Figure 2a). Except for the HK II protein content in the m. quadriceps femoris, COX I and HK II protein contents were significantly increased in the exercise wheel group (Figure 2b). Exercise wheel running resulted in a significant decrease in breathing frequency, but tidal volume and minute volume were equal between both experimental groups (Figure 2c).

### Effect of exercise on symptom severity score and lung bacteriology

To determine that the lungs were selectively targeted by nasal inoculation, intranasal administration of radio-labelled peptide solution (50 μl) was performed as has been described, in a separate group of mice (data not shown) [18]. The mean percentage of radioactive particles detected in the lungs (relative to the control fluid) was 71% (range 61–76%). No radio-active particles were detected in the stomachs of the mice, indicating that the inocula were specifically delivered to the lungs without being swallowed.

**Figure 3:**
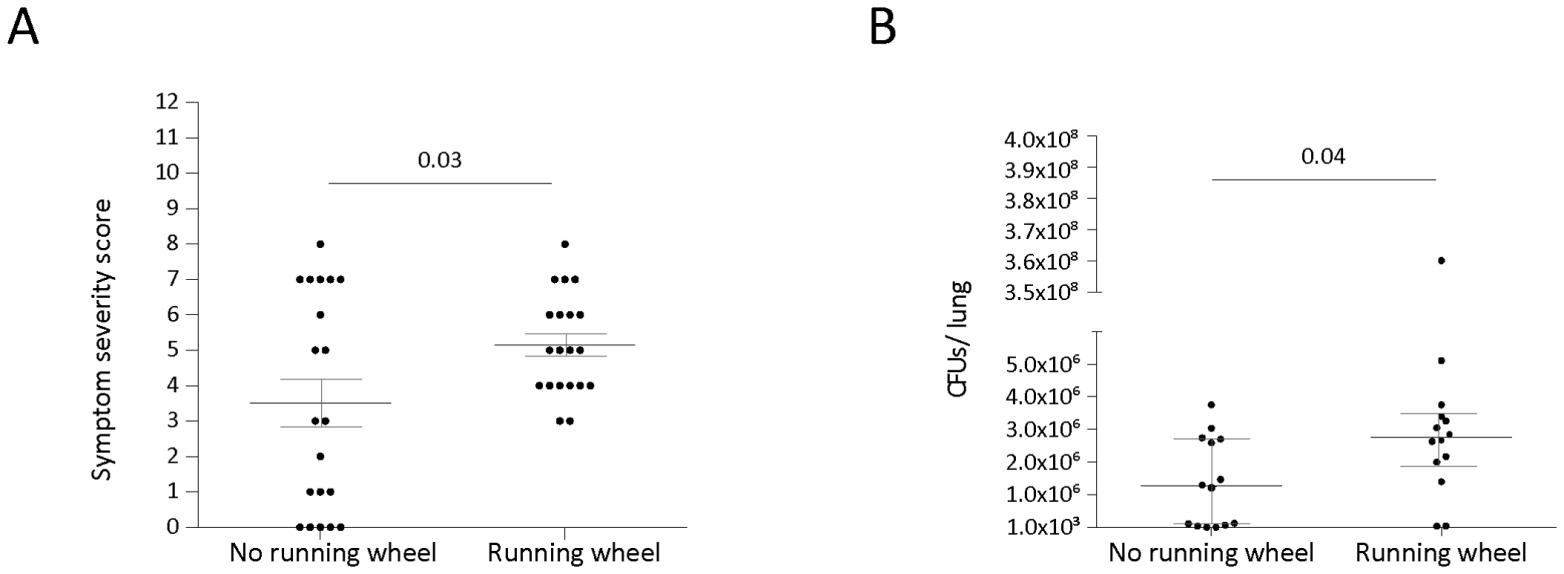
Effect of exercise on symptom severity score and bacterial load in the lung following intranasal inoculation with *P. aeruginosa.* Each dot represents one mouse. **A.** Symptom severity score (Mean ± SEM) 16 hours following intranasal inoculation with P. aeruginosa (n = 20 per group). **B**. Amount of colony forming units per lung (Median ± IR) following intranasal inoculation with P. aeruginosa (n = 14 per group). Inoculation doses was 5×10^6^ CFUs/50 μL.

To investigate whether regular exercise affects *P. aeruginosa* pulmonary infection susceptibility, post-infection symptom severity score and lung bacteriology were determined. Mice in the running wheel group had a significantly higher symptom severity score, suggesting more severe illness, 16 hours after intranasal inoculation with *P. aeruginosa* (Figure 3a). Furthermore, mice in the exercise wheel running group had a significantly higher amount of *P. aeruginosa* CFU in their lungs (Figure 3b).

### Effect of exercise on the phagocytic capacity of phagocytes

**Figure 4:**
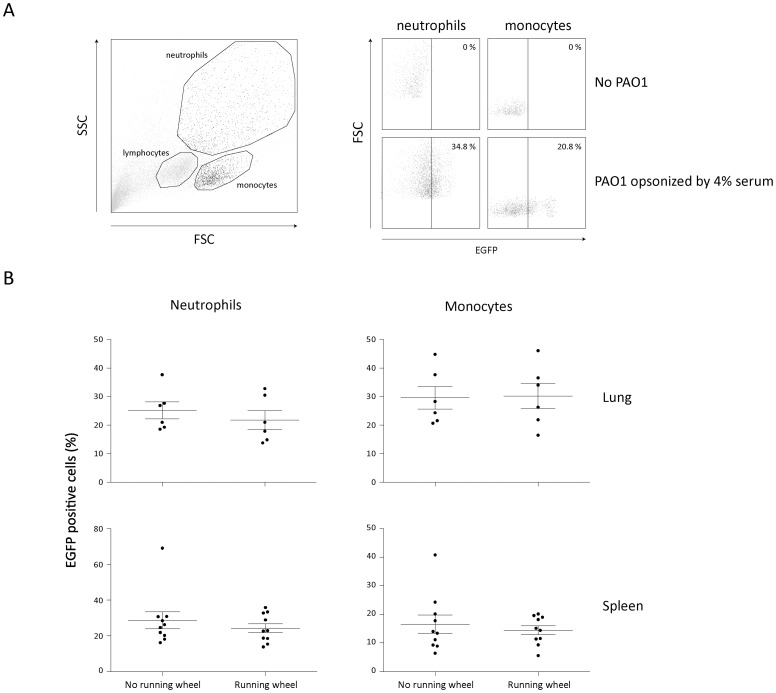
Effect of exercise on capacity of phagocytes to take up *P. aeruginosa.* **A.** Monocytes and neutrophils were gated by forward and side scatter (left panel). Representative dotplots of monocytes (left panel) and neutrophils (right panel) incubated with (lower panel) or without (upper panel) EGFP-labeled P. aeruginosa and selection of EGFP-positive cells. Bacteria were opsonized with 4% mouse serum. **B.** Phagocytic capacity of neutrophils and monocytes (Mean ± SEM) isolated from the spleen (n = 6 per group) and lungs (n = 10 per group). Each dot represents one mouse.

The phagocytic capacity of monocytes and polymorphonuclear leukocytes (neutrophils) was determined to investigate whether regular exercise affects the phagocytic capacity of phagocytes and whether a change in these innate immune functions could be associated with the changes in lung bacteriology. The phagocytic capacity was determined using flow cytometry by analyzing uptake of EGFP-labelled *P. aeruginosa* by monocytes and neutrophils after 30 minutes of co-culture (Figure 4a shows representative examples). The capacity of monocytes and neutrophils to phagocytose *P. aeruginosa*, 16 hours post-infection, was not affected by regular exercise (Figure 4b).

## Discussion

The aim of this study was to investigate whether regular voluntary exercise prior to a pulmonary infection with *P. aeruginosa* in mice affects lung bacteriology, sickness severity and phagocyte immune function. We observed that exercised mice had more severe illness and a higher *P. aeruginosa* infection loads in the lungs. The phagocytic capacity of monocytes and neutrophils from spleen and lungs was not affected 16 hours post-infection. Collectively, these data indicate that regular voluntary exercise can enhance susceptibility to a bacterial pulmonary infection.

We are the first investigating the effect of regular exercise on bacterial infection load. Limited data are available showing that regular exercise leads to a reduced viral infection load and associated morbidity and mortality in animals [10]–[12] and a reduced upper respiratory tract infection frequency in humans [13]. The contrasting findings of this study may result from differences in microorganism-infection model (virus versus bacteria) or animal model. Inflammatory responses are in general greater in female compared to male Balb/c mice. Use of other mice strains may lead to complete different results. Additionally, future research should focus on longer follow-up times post-infection, since different kinetics at different time-points post-infection may lead to different results. Different *P. aeruginosa* strains should therefore be used, such as chronic (non-)mucoid *P. aeruginosa* strains (e.g. NH57388) [23], which allows longer follow-up times due to their lower lethality. Furthermore, the exercise modality used in our study had a voluntary character, which may induce completely different effects on infection susceptibility compared to involuntary treadmill running used in the previous studies [10]–[12].

Voluntary exercise wheel running was chosen as an exercise model, since a standardized treadmill exercise protocol leads to stress responses due to its involuntary character, which may therefore be a potential study confounder [24]. It has been demonstrated that voluntary exercise wheel running leads to similar skeletal muscle adaptations as standardized involuntary moderate treadmill running [25], supporting its use as effective exercise modality to induce training effects in mice. Average running distance covered by the mice in our study was comparable to what was found in other studies [26]; [27]. Furthermore, we showed that 4 weeks of voluntary exercise wheel running induced an aerobic training effect on skeletal muscle enzyme content, which was comparable to what was shown for HKII [28]; [29] and COXI [30] by previous studies. Taken together, these data suggest that voluntary exercise wheel running in our study was effective to induce skeletal muscle adaptations and is representative for moderate exercise. Furthermore, this is the first study that defines mouse phagocyte function at the level of individual cells in response to exercise and infection using a novel assay. The data show that exercise and infection does not modulate intrinsic phagocyte function, which we believe is relevant and novel to the field.

A change in the phagocytic capacity of monocytes and neutrophils might be an explanation for the increased exercise-induced bacterial infection susceptibility. However, although a higher sickness severity and lung bacteriology were measured after regular voluntary exercise, we showed that the capacity of neutrophils and monocytes to phagocytose *P. aeruginosa* was unaffected 16 hours post-infection. Whether innate immune function is directly affected by regular exercise has been poorly investigated by others and contrary results have been published. Regular intense exercise leads to a reduced capacity of neutrophils to phagocytose unopsonized latex beads and produce superoxide anions [31], whereas this was not affected [31] or improved [32] by regular moderate exercise. In our phagocytosis assay opsonized live bacteria and whole blood cells in co-culture were used, which allows bacteria-cell and cell-cell interactions that may modulate bacterial defense mechanisms and represents the in vivo situation better. This novel assay allows to define mouse phagocyte function at the level of individual cells in response to exercise and infection and is therefore of additional value to the field. Altogether, the role of exercise-induced changes in innate immune function remains unclear. Effects are probably dependent on the exercise modality and animal model used. Furthermore, more studies are needed addressing the role of exercise in differential innate immune defense mechanisms. For future research, a separate group of mice, not infected by *P. aeruginosa*, should be included. This will help unravel exercise-induced changes in immune defense mechanisms that may not be visible anymore 16 hours post-infection.

We observed no effect of regular voluntary exercise on the phagocytic capacity of neutrophils and monocytes, however other innate immune functions, such as the killing capacity of phagocytes or defensin levels in the lung, might be affected. Furthermore, it has been reported that moderate regular exercise leads to reduced circulating levels of cells with an activated phenotype, indicated by increased anti-inflammatory regulatory T cell (Treg) [33] and reduced inflammatory (CD16^+^ high) monocytes levels [8]; [34]–[37]. These preferential changes in circulating subsets towards cells with a reduced activated phenotype might lead to reduced antigen responsiveness, which may lead to a reduced antigen clearance and increased disease frequency. Unfortunately, changes in cell population numbers have not been estimated in our study.

It may be possible to extrapolate data to other bacteria that more commonly lead to pulmonary infections, such as *Staphylococcus aureus*, *Streptococcus pneumoniae* or *Hemophilus influenzae*. However, this requires more research, since differences in immunological responses to different bacteria and dissimilarities in virulence factors may lead to different results.

Collectively, our data showed that voluntary moderate exercise can enhance bacterial (*P. aeruginosa*) infection susceptibility with perseverance of the phagocytic capacity of monocytes and neutrophils 16 hours post-infection. However, it requires further research to explore innate immune defense mechanisms at other time-points than 16-hours post-infection and measurement of other determinants that may be involved. Whether cell numbers or other innate immune functions, such as the killing capacity, were affected, has to be studied.

## Conclusion

Although regular moderate exercise has many health benefits, healthy mice show increased bacterial (*P. aeruginosa*) infection load and symptoms, after regular voluntary exercise, with perseverance of the phagocytic capacity of monocytes and neutrophils. Whether patients, suffering from bacterial infectious diseases, should be encouraged to engage in exercise and physical activities with caution, requires further research.
